# A nomogram for predicting the risk of treatment failure of roxadustat in peritoneal dialysis with renal anemia

**DOI:** 10.1038/s41598-024-58289-z

**Published:** 2024-04-01

**Authors:** Jiangqing Fan, Wenpu Lei, Lulu Wang, Weihong Ge

**Affiliations:** 1 Department of Pharmacy, Nanjing Drum Tower Hospital, School of Basic Medicine and Clinical Pharmacy, China Pharmaceutical University, Nanjing, China; 2 Department of Pharmacy, Nanjing Drum Tower Hospital, Affiliated Hospital of Medical School, Nanjing University, Nanjing, China; 3 Hunan Provincial Key Laboratory of the Research and Development of Novel Pharmaceutical Preparations, The “Double-First Class” Application Characteristic Discipline of Hunan Province (Pharmaceutical Science), Changsha Medical University, Changsha, China

**Keywords:** Peritoneal dialysis, Renal anemia, HIF-inhibitor, Nomogram, Diseases, Risk factors

## Abstract

The determinants of roxadustat treatment failure in renal anemia remain elusive. This study sought to develop a nomogram for predicting the risk of treatment failure of roxadustat in peritoneal dialysis (PD) with renal anemia. A retrospective cohort analysis from January 1, 2019, to January 31, 2023, included 204 PD patients with renal anemia, stratified by attainment group (Hb ≥ 110 g/L, n = 103) or non-attainment (Hb < 110 g/L, n = 101) within 1 year treatment. Univariate and multivariate Cox proportional hazards regressions were employed to ascertain predictive factors and construct the nomogram. Nomogram efficacy was evaluated via C-index, time-dependent ROC, calibration plots, and decision curve analysis, with internal validation via tenfold cross-validation and 1000 bootstrap resampling iterations. The study identified PD duration, serum transferrin, cardiovascular comorbidities, and stains as significant predictors. The nomogram demonstrated moderate discrimination at 6 months (AUC: 0.717) and enhanced predictive accuracy at 12 months (AUC: 0.741). The predicted and actual risk probabilities were concordant, with clinical net benefits observed at six-month (8 to 53%) and twelve-month (27 to 84%) risk thresholds. This nomogram is a valuable tool for effectively predicting non-attainment risk and facilitating personalized management of renal anemia in PD patients treated with roxadustat.

## Introduction

Renal anemia is a prevalent complication of chronic kidney disease (CKD), constituting an independent risk factor for cardiovascular events and mortality^1^. This condition arises primarily from erythropoietin (EPO) deficiency and disrupted iron metabolism due to compromised renal function, with the prevalence and severity of anemia escalating as kidney disease progresses^1^.

The standard treatment paradigm for renal anemia combines erythropoiesis-stimulating agents (ESAs) with iron agency. On December 18, 2018, the National Medical Products Administration of China granted approval for roxadustat capsules, a Class One innovative drug, for the treatment of anemia in dialysis patients with CKD^2,3^. Oral roxadustat capsules are the first small molecule hypoxia‐inducible factor prolyl hydroxylase inhibitor for the treatment of renal anemia^3^. Clinical evidence indicates that roxadustat induces a modest and transient increase in endogenous erythropoietin, which is well-tolerated and comparable to ESAs in terms of efficacy and cardiovascular safety^4,5^. Furthermore, the oral administration route enhances patient compliance, offering a new therapeutic avenue for renal anemia management.

Renal anemia is highly prevalent among end-stage kidney disease (ESKD) patients undergoing dialysis, with over 90% affected^6^. In the peritoneal dialysis (PD) population, the prevalence is approximately 61.2%^7^. In the Asia–Pacific region, 82–96% of anemic PD patients receive ESAs^8^. In China, the hemoglobin (Hb) attainment rate for renal anemia treatment is suboptimal, with approximately 60% of anemic dialysis patients failing to achieve Hb target levels^7–9^. The accumulation of uremic toxins and inflammation in PD patients exacerbates renal anemia, elevates the risk of cardiovascular events and mortality, and significantly impacts patient quality of life^10,11^. However, predictive models for the risk of non-attainment of Hb target levels during roxadustat treatment are lacking. Therefore, we aimed to investigate the risk factors associated with roxadustat treatment failure in PD patients with renal anemia and to develop a predictive model for the risk of non-attainment of Hb target levels after roxadustat treatment. This model will aid in the personalized management of nephrogenic anemia therapies.

## Methods

### Study design and participants

This single-center, retrospective study analyzed data from January 1, 2019, to January 31, 2023, at the Department of Nephrology, Nanjing Drum Tower Hospital, focusing on patients with PD and renal anemia treated with roxadustat. The study excluded patients receiving hemodialysis, blood transfusions, ESAs, or iron agency alone, as well as those with incomplete clinical data or insufficient 1 year follow-up. Inclusion criteria encompassed patients with stage 5 CKD and renal anemia undergoing PD and treated with roxadustat. This study was approved by the Medical Ethics Committee of Nanjing Drum Tower Hospital (Ethics Approval Number: 2023-118-01), written informed consent was obtained from all participants before data collection, and all procedures adhered to the principles of the Declaration of Helsinki.

### Therapeutic intervention

Roxadustat capsules, with a specification of 20 mg/50 mg per tablet (national drug approval number H20180024), were administered orally three times weekly, with dosages adjusted from 50 mg, 70 mg, 100 mg, and 120 mg based on patient's follow-up Hb levels. The initial dose was determined by body weight, with 100 mg per administration for patients weighing less than 60 kg and 120 mg per administration for those weighing 60 kg or more. Transitioning patients from ESA to roxadustat therapy received 70 mg per administration if their rHuEPO was less than 4500 U/week, and 100 mg per administration if it was 4500 U/week or more. Physicians adjusted the dosage considering the patient's follow-up Hb level and individual factors.

### Data collection

Basic demographic and clinical data, including follow-up Hb levels within 1 year, were collected from the Health Information System (HIS) and telephone interviews with participating patients. The demographic profile included age, gender, body mass index (BMI), and smoking status. Clinical data comprised PD duration, initial dosage of roxadustat, the primary renal disease, and comorbidities such as diabetes mellitus, hypertension, cardiovascular diseases (including coronary artery disease, cerebrovascular accident, heart failure, cardiomyopathy, arrhythmias, valvular heart disease, myocarditis, and peripheral arterial disease), infections, liver cirrhosis, and gastrointestinal bleeding. Additional data included concomitant medications (iron agency, phosphorus binders, and statins) and laboratory parameters like reticulocyte count (Ret), Hb, erythropoietin (EPO), transferrin saturation (TSAT), serum ferritin (SF), serum transferrin (sTf), total carbon dioxide (TCO2), platelet-to-lymphocyte ratio (PLR), neutrophil-to-lymphocyte ratio (NLR), serum phosphate, brain natriuretic peptide (BNP), alkaline phosphatase (ALP), albumin (ALB), serum creatinine (Scr), C-reactive protein (CRP), estimated glomerular filtration rate (eGFR), total cholesterol (TC), triglycerides (TG), and low-density lipoprotein cholesterol (LDL). Follow-up data collection concluded on September 1, 2023.

### Clinical efficacy evaluation and groups

This study evaluated the clinical efficacy of roxadustat in treating anemia in patients in alignment with the "Chinese clinical practice guidelines for the diagnosis and treatment of nephrogenic anemia^7^ and the "Chinese expert consensus on the treatment of renal anemia"^12^. The evaluation was based on Hb levels throughout the 1 year follow-up period. The Cox proportional hazards regression model was employed, using time-related events as the outcome indicator. The outcome variable was defined as the failure to achieve the target Hb levels at the 1 year follow-up. The time variable was delineated as the period from the commencement of roxadustat treatment until the point at which the target Hb levels were not attained. Cases with follow-up Hb < 110 g/L were included in the non-attainment Hb target levels group, and the others were included in the attainment Hb target levels group.

### Statistical analysis

Data cleaning and preparation were conducted in SPSS 26.0, with all hypothetical predictors encoded as categorical variables and quantified using case counts and percentiles. The development, visualization, and validation of Cox proportional hazards regression model were carried out using R 4.2.3 software. Sample size estimation was based on the 10 Events Per Variable (10 EPV) method, and cases with missing data were systematically excluded. Data import was facilitated by the "foreign" package in R. Univariate and multivariate analyses, as well as the fitting of the Cox proportional hazards regression model, were conducted using the "survival" package and the 'coxph' function. Variables with a *p*-value less than 0.2 in the univariate analysis were considered for inclusion in the multivariate Cox proportional hazards regression model. Predictive variables selection was performed through stepwise regression analysis, guided by the Akaike Information Criterion (AIC), with a preference for models exhibiting the lowest AIC values. The final model included variables with *p*-values greater than 0.05 if they contributed to the minimal AIC. The "rms" package was employed to develop a nomogram for predicting the probability of treatment failure with roxadustat. The "survivalROC" package and the 'predict' function were used to estimate the predicted probabilities at 6 and 12 months post-treatment. Model discrimination was evaluated by fitting survival ROC curves at these time points using the Kaplan–Meier (KM) method, with higher Area Under the Curve (AUC) values indicating superior discrimination. Calibration plots were generated using the 'calibrate' function from the "rms" package to assess the model's accuracy. Clinical utility was evaluated through Decision Curve Analysis (DCA), performed with the "stdca" package. The 'plot' function facilitated the visualization of these curves. Internal validation was conducted using the bootstrap self-sampling method, with 1,000 resampling iterations, employing the 'boot' function.

### Ethics approval and consent

This study received approval from the Medical Ethics Committee of Nanjing Drum Tower Hospital (Ethics Approval Number: 2023–118-01). Written informed consent was obtained from each participant prior to data collection, and all procedures strictly adhered to the principles of the Declaration of Helsinki.

## Results

### General information of study subjects

**Figure 1:**
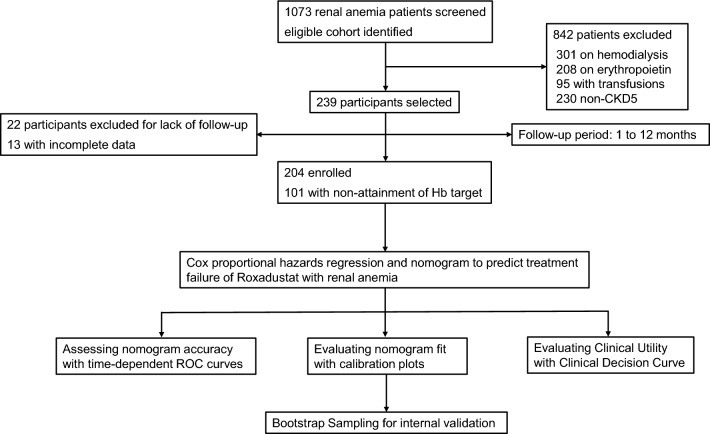
Study flowchart.

This retrospective study reviewed a total of 1,073 patients with renal anemia at Nanjing Drum Tower Hospital from January 1, 2019, to January 31, 2023. Following the application of inclusion and exclusion criteria, 301 patients receiving hemodialysis, 230 with non-stage 5 CKD, 208 on ESAs, and 95 who had undergone blood transfusions were excluded. A total of 239 patients met the criteria for inclusion. Of these, 22 were lost to follow-up, and data were incomplete for an additional 13 patients, resulting in a final cohort of 204 PD patients with renal anemia. Among these, 101 patients failed to achieve their target hemoglobin levels after 1 year of roxadustat treatment, as depicted in Fig. 1. The age, gender, initial dose of roxadustat, primary renal disease, and concurrent use of iron agency showed no significant differences between the attainment and non-attainment groups. The general characteristics of the study population are detailed in Table 1. Significant differences in baseline data for PD duration, prevalence of cardiovascular disease, and statin use were observed between groups (*p* < 0.05), as presented in Table 2.

**Table 1:** Demographic and clinical characteristics of the study participants.

Variables	Overall (n = 204)	non-attainment group (N = 101)	attainment group (N = 103)	*P* value
Age				0.164
< 53	85(41.7%)	48 (47.5%)	37 (35.9%)	
≥ 53	119(58.3%)	53(52.5%)	66(64.1%)	
Gender				0.962
Female	87(42.6%)	46(45.5%)	41(39.8%)	
Male	117(57.4)	55(54.5%)	62(60.2%)	
Roxadustat dosage				0.092
50 mg	25(12.3%)	9(8.9%)	16(15.5%)	
70 mg	70(34.3%)	34(33.7%)	36(35%)	
100 mg	107(52.5%)	56(55.4%)	51(49.5%)	
120 mg	2(1%)	2(2%)	–	
Primary renal disease				0.495
Diabetes nephropathy	61(29.9%)	31(30.7%)	30(29.1%)	
Hypertensive kidney disease	65(31.9%)	25(24.8%)	40(38.8%)	
Chronic nephritis	72(35.3%)	39(38.6%)	33(32%)	
Other types	6(2.9%)	6(5.9%)		
Combined iron agency				0.256
Yes	69(33.8%)	38(37.6%)	31(30.1%)	
No	135(66.2%)	63(62.4%)	72(69.9%)

**Table 2:** Univariate and multivariate analysis of predictors for roxadustat treatment failure in patients with renal anemia.

Basic characteristics	Univariate analysis			Multivariate analysis		
	HR^a^	95% CI^b^	*P* value	HR	95% CI	*P* value
Demographic data						
Body mass index (< 25 kg/m^2^ vs. ≥ 25 kg/m^2^)	0.89	0.73–1.73	0.583			
Smoking history (no vs. yes)	1.08	0.51–1.7	0.802			
PD duration (vs. ≥ 1 year)						
< 1 year	1.22	0.56–2.66	0.621	1.10	0.50–2.40	0.821
Initial PD catheter	2.15	1.27–3.65	0.005	2.49	1.45–4.28	0.0009
Laboratory tests						
Reticulocyte (≤ 1.5% vs. > 1.5%)	1.23	0.52–1.28	0.373			
Hemoglobin (< 100 g/L vs. ≥ 100 g/L)	0.77	0.63–2.67	0.486			
Erythropoietin (vs. > 29 mIU/ml)						
4.3–29 mIU/ml	0.92	0.50–1.27	0.718			
< 4.3 mIU/ml	1.36	0.91–1.72	0.073			
Transferrin saturation (< 20% vs. ≥ 20%)	0.84	0.78–1.79	0.423			
Serum ferritin (< 100 ug/L vs. ≥ 100 ug/L)	0.74	0.84–2.18	0.210			
Serum transferrin (< 2 g/L vs. 2–3.6 g/L)	1.45	0.46–1.03	0.071	0.68	0.46–1.02	0.065
Blood total carbon dioxide (< 22 mmol/L vs. ≥ 22 mmol/L)	0.73	0.92–2.04	0.116			
PLR (< 180 vs. ≥ 180)	1.34	0.5–1.11	0.144			
NLR (< 3.1 vs. ≥ 3.1)	1.38	0.47–1.12	0.150			
Serum phosphate (1.6–0.96 mmol/L vs. > 1.62 mmol/L)	1.31	0.51–1.14	0.188			
Alkaline phosphatase(vs. > 185 U/L)						
47–185 U/L	0.50	0.20–1.26	0.139			
< 47 U/L	0.70	0.40–1.21	0.207			
BNP (5–100 pg/mL vs. > 100 pg/mL)	0.76	0.87–1.97	0.191			
Albumin (< 40 ug/L vs. ≥ 40 ug/L)	1.13	0.41–1.92	0.762			
Scr (44–106 umol/L vs. > 106 umol/L)	1.00	0.37–2.68	0.998			
CRP (< 8 mg/L vs. ≥ 8 mg/L)	0.93	0.71–1.65	0.719			
eGFR (< 90 ml/min/1.73–^2^ vs. ≥ 90 ml/min/1.73–^2^)	0.00	0-Inf	0.995			
TC(vs. > 5.72 mmol/L)						
2.9–5.72 mmol/L	0.87	0.46–1.64	0.665			
< 2.9 mmol/L	0.75	0.49–1.11	0.159			
TG(vs. > 1.7 mmol/L)						
0.56–1.7 mmol/L	2.18	0.54–8.92	0.277			
< 1.7 mmol/L	0.68	0.29–1.57	0.369			
LDL(vs. > 3.1 mmol/L)						
1.89–3.1 mmol/L	0.88	0.57–1.34	0.537			
< 1.89 mmol/L	0.87	0.62–1.22	0.416			
Comorbidities (no vs. yes)						
Diabetes mellitus	1.01	0.66–1.48	0.955			
Infection	0.77	0.86–1.98	0.206			
Hypertension	1.18	0.41–1.75	0.651			
Cardiovascular diseases	1.65	0.4–0.92	0.019	0.63	0.40–0.97	0.038
liver cirrhosis	1.33	0.33–1.72	0.499			
Gastrointestinal bleeding	0.79	1.46–3.45	0.648			
Combined medication (no vs. yes)						
Iron agency	1.05	0.64–1.43	0.814			
PHOSPHATE-Lowering agents	1.02	0.67–1.46	0.937			
Stains	0.57	1.02–3.01	0.041	1.63	0.91–2.92	0.097

*HR* hazard ratio, *CI* confidence interval, *PD* peritoneal dialysis, *PLR* platelet to lymphocyte ratio, *NLR* neutrophil to lymphocyte ratio, *BNP* brain natriuretic peptide, *Scr* Serum creatinine, *CRP* C-reactive protein, *eGFR* estimated glomerular filtration rate, *TC* total cholesterol, *TG* triglyceride, *LDL* low density lipoprotein cholesterol.

### Influencing factors for non-attainment of hemoglobin target levels following roxadustat treatment

In the univariate analysis, fourteen variables with a *p*-value less than 0.2 were identified as potential predictors for inclusion in the multivariate analysis. These predictors included PD duration, cardiovascular disease, statins, EPO, TC, sTf, roxadustat initial dosage, TCO_2_, PLR, NLR, age, hemophosphates, ALP, and BNP. The dependent variable was the Hb value at follow-up after roxadustat treatment within 1 year. A multivariate Cox proportional hazards regression analysis was conducted to further elucidate the influence of these independent factors on the efficacy of roxadustat in managing renal anemia in PD patients. The results indicated that PD duration and cardiovascular disease were statistically significant between the two groups, as detailed in Table 2.

### Development of a risk nomogram for roxadustat treatment failure in peritoneal dialysis patients with renal anemia

**Figure 2:**
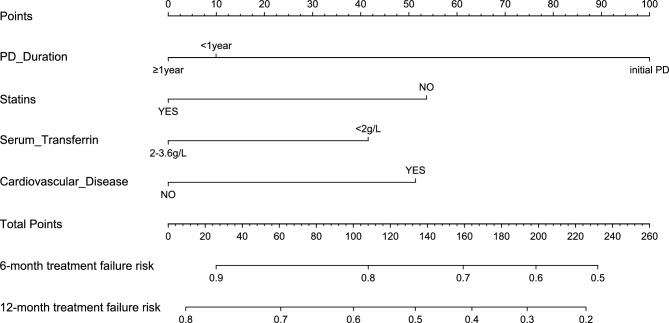
Nomogram for predicting treatment failure risk of roxadustat in peritoneal dialysis patients with renal anemia.

The risk nomogram was constructed utilizing variables identified by the multivariate analysis with the smallest AIC values: PD duration, sTfR, cardiovascular disease, and statins as the final predictive factors (minimal AIC = 881.43, depicted in Fig. 2). The predictive model, incorporating these factors, demonstrated a good fit, as evidenced by the likelihood ratio test (*p* = 8e-05), Wald test (*p* = 1e-04), and logrank test (*p* = 1e-04).

For illustrative purposes, consider an end-stage kidney disease (ESKD) patient with the following parameters: a PD duration of 2 years (0 points), a history of heart disease (53 points), sTf at 1.5 g/L (41 points), and statin use (0 points). The cumulative score, accounting for these parameters, totals 94 points. The estimated risk of treatment failure following roxadustat treatment for this patient is approximately 82% at six months and 61% at 12 months.

### Evaluation of prediction efficiency for the nomogram

**Figure 3:**
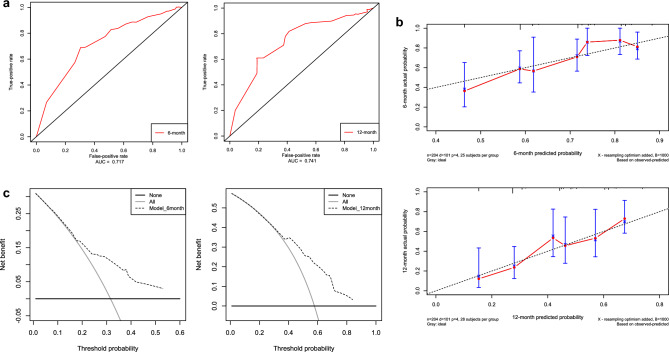
Assessment of nomogram prediction efficiency and clinical utility. (**a**) ROC curves for six- and twelve-month treatment failure predictions. (**b**) Calibration curves for six- and twelve-month Hb target non-attainment risks. (**c**) Clinical decision curve analysis for Hb target attainment.

The nomogram demonstrated an AUC of 0.717 for six-month and 0.741 for twelve-month roxadustat treatments, indicating its predictive accuracy (Fig. 3a). The calibration curve, derived from 1,000 bootstrap self-sampling iterations, revealed that the nomogram's predicted risk curve closely approximated the actual risk curve at both six and 12 months, with only minor deviations, suggesting a well-fitted model (Fig. 3b).

### Clinical utility of the nomogram for predicting roxadustat treatment failure

The clinical decision curve analysis revealed that the nomogram provided effective assessment of the probability of non-attainment of Hb target levels following 6 months of roxadustat treatment, within a risk threshold of 8 to 53%, leading to a positive clinical net benefit (Fig. 3c). Furthermore, the nomogram indicated that for risk thresholds ranging from 27 to 84%, the prediction of non-attainment of Hb target levels over 12 months with roxadustat treatment could yield a net clinical benefit (Fig. 3c).

## Discussion

Renal anemia is a prevalent condition among patients undergoing PD, often associated with a suboptimal rate of treatment success^13^. The early identification of factors that contribute to the non-attainment of Hb target levels is essential for improving the quality of life and potentially delaying the progression of the disease. This study harnessed baseline data from patients with PD and renal anemia before the initiation of roxadustat therapy to construct a predictive model that anticipates treatment outcomes. This model is designed to facilitate personalized treatment approaches for patients with renal anemia, enhancing the clinical management of roxadustat therapy.

The current literature on predictive models for therapeutic interventions involving roxadustat is limited, highlighting a need for further research in this area. Our study has developed a nomogram to predict the risk of non-attainment of Hb target levels following roxadustat treatment, which incorporates cardiovascular diseases, PD duration, sTf, and statins as predictive factors. Despite the inclusion of variables with *p*-values exceeding the standard threshold of 0.05, the model exhibited optimal AIC, good overall fit, and adequate predictive performance. The AUC values for the nomogram in predicting the risk of non-attainment of Hb target levels after six and 12 months of roxadustat treatment were greater than 0.7, indicating that the model has good predictive efficacy. The evaluation of the model's goodness-of-fit revealed a strong alignment between the predicted and actual risk probabilities, further validating its reliability. The observed rate of non-attainment of Hb target levels during roxadustat treatment was 49.5%. The clinical decision curve analysis at this threshold for both six and 12 months of treatment positioned the nomogram above the pre-intervention and intervention lines, which signifies the model's clinical utility and practical applicability. This finding suggests that the nomogram can effectively guide clinical decision-making and enhance the personalized management of renal anemia in patients undergoing PD.

Our nomogram has identified that PD duration, cardiovascular diseases, sTf, and statin therapy at baseline are significantly associated with the risk of non-attainment of Hb target levels in patients with renal anemia treated with roxadustat. These predictive factors are corroborated by existing literature on renal anemia treatment, aligning with our study's findings. Our findings suggest that cardiovascular disease is a significant independent risk factor for suboptimal renal anemia treatment response. This is in line with the findings of Kidanewold et al., who observed a significant correlation between cardiovascular disease and anemia in CKD patients^14^. Patients with shorter PD duration exhibit a higher risk of not achieving Hb target levels, possibly due to the necessity for regular evaluation and monitoring in long-term PD patients, which may contribute to the reduced risk of treatment failure in this cohort. Transferrin primarily transports serum iron to target organs to facilitate red blood cell production, serving as a crucial regulator of iron homeostasis^15,16^. In clinical practice, transferrin levels and transferrin receptors serve as markers for various diseases^17,18^. Beguin Y's study supports this by showing that baseline transferrin receptor (TfR) levels can predict the response to recombinant human erythropoietin (rHuEPO)^19^. Our research further demonstrates that patients with renal anemia who are on statins experience a 63% reduction in the risk of non-attainment of Hb target levels following roxadustat treatment, compared to those not on statins. This is consistent with evidence suggesting that statins can ameliorate renal anemia, mitigate ESA hyporesponsiveness, and reduce inflammation^20,21^.

This retrospective study aimed to evaluate the risk of treatment failure associated with roxadustat in patients with PD and renal anemia. Compliance with the treatment regimen was ascertained through a combination of healthcare information system (HIS) follow-up records and telephone interviews with participating patients, providing insights into medication adherence over the study period. While the construction of our nomogram from a retrospective dataset from a single center and its validation through 1,000 iterations of bootstrap self-sampling represent a methodological strength, we acknowledge the inherent limitations in the lack of external validation for the model. The reliance on a single-center dataset introduces a degree of institutional bias that may affect the broader applicability of our model. Consequently, we encourage future research to advance our preliminary work by incorporating multicenter studies with larger and more diverse patient populations, which would enhance the nomogram's predictive accuracy and its applicability to varied patient groups.

## Conclusion

The developed nomogram effectively predicts the risk of non-attainment of hemoglobin target levels in peritoneal dialysis patients with renal anemia undergoing roxadustat therapy. The model incorporates critical parameters such as cardiovascular comorbidities, PD duration, sTf, and statins, offering a valuable visual aid for healthcare professionals. This nomogram is instrumental in the early detection of patients with renal anemia who may exhibit suboptimal responses to roxadustat, thereby facilitating more targeted and effective clinical interventions. Furthermore, the nomogram provides a foundation for the development of individualized pharmacotherapeutic strategies for patients with renal anemia, contributing to the optimization of roxadustat treatment in the context of abdominal dialysis and renal anemia.

## Data Availability

All data generated or analyzed during this study are included in this article. Further inquiries can be directed to the corresponding author.

## References

[1] Fishbane S, Coyne DW (2020). How I treat renal anemia. Blood.

[2] Dhillon S (2019). Roxadustat: First global approval. Drugs.

[3] National Medical Products Administration. *New drug Roxadustat capsules for the treatment of renal anemia granted market approval*. https://www.nmpa.gov.cn/yaopin/ypjgdt/20181218092001170.html (2018).

[4] Sugahara M, Tanaka T, Nangaku M (2022). Future perspectives of anemia management in chronic kidney disease using hypoxia-inducible factor-prolyl hydroxylase inhibitors. Pharmacol. Ther..

[5] Locatelli F, Del Vecchio L (2022). Hypoxia-inducible factor-prolyl hydroxyl domain inhibitors: From theoretical superiority to clinical noninferiority compared with current ESAs?. J. Am. Soc. Nephrol..

[6] Zhang L (2012). Prevalence of chronic kidney disease in China: A cross-sectional survey. Lancet.

[7] Chinese medical Physicians association nephrologist branch nephrology guidelines working group (2021). Clinical practice guideline for the diagnosis and treatment of renal anemia in China. Natl. Med. J. China.

[8] Li PKT (2021). Anemia management in peritoneal dialysis: Perspectives from the asia pacific region. Kidney Med..

[9] Zhou QG (2012). Current pattern of Chinese dialysis units: A cohort study in a representative sample of units. Chin. Med. J..

[10] Babitt JL, Lin HY (2012). Mechanisms of anemia in CKD. J. Am. Soc. Nephrol..

[11] Lee SW (2017). Serum hepcidin may be a novel uremic toxin, which might be related to erythropoietin resistance. Sci. Rep..

[12] Nephrology Professional Committee of Chinese Research Hospital Association (2022). The expert consensus on the treatment for renal anemia with Roxadustat in China. Natl. Med. J. China.

[13] Perlman RL (2019). International anemia prevalence and management in peritoneal dialysis patients. Perit. Dial. Int..

[14] Kidanewold A, Woldu B, Getie A, Enawgaw B (2022). Anemia and its predictors among adult non-dialysis chronic kidney disease patients in Southern Ethiopia: A cross-sectional study. Curr. Med. Res. Opin..

[15] Boshuizen M (2017). Therapeutic use of transferrin to modulate anemia and conditions of iron toxicity. Blood Rev..

[16] Bartnikas TB (2012). Known and potential roles of transferrin in iron biology. Biometals.

[17] Günther F (2021). Association of serum soluble transferrin receptor concentration with markers of inflammation-analysis of 1001 patients from a tertiary rheumatology center. J. Rheumatol..

[18] Shen Y (2018). Transferrin receptor 1 in cancer: A new sight for cancer therapy. Am. J. Cancer Res..

[19] Beguin Y (1993). Early prediction of response to recombinant human erythropoietin in patients with the anemia of renal failure by serum transferrin receptor and fibrinogen. Blood.

[20] Koc M (2011). Statin use is associated with lower inflammation and erythropoietin responsiveness index in hemodialysis patients. Hemodial. Int..

[21] Tsai MH (2022). The Effect of statin on anemia in patients with chronic kidney disease and end-stage kidney disease: A systematic review and meta-analysis. J. Pers. Med..

